# Implication of microRNA regulation in *para*-phenylenediamine-induced cell death and senescence in normal human hair dermal papilla cells

**DOI:** 10.3892/mmr.2015.3487

**Published:** 2015-03-13

**Authors:** OK-KYU LEE, HWA JUN CHA, MYUNG JOO LEE, KYUNG MI LIM, JAE WOOK JUNG, KYU JOONG AHN, IN-SOOK AN, SUNGKWAN AN, SEUNGHEE BAE

**Affiliations:** 1 Korea Institute for Skin and Clinical Sciences and Molecular-Targeted Drug Research Center, Konkuk University, Seoul 143-701, Republic of Korea; 2 Department of Dermatology, Konkuk University School of Medicine, Seoul 143-729, Republic of Korea

**Keywords:** *para*-phenylenediamine, human dermal papilla cells, microRNA

## Abstract

*Para*-phenylenediamine (PPD) is a major component of hair coloring and black henna products. Although it has been largely demonstrated that PPD induces allergic reactions and increases the risk of tumors in the kidney, liver, thyroid gland and urinary bladder, the effect on dermal papilla cells remains to be elucidated. Therefore, the current study evaluated the effects of PPD on growth, cell death and senescence using cell-based assays and microRNA (miRNA) microarray in normal human hair dermal papilla cells (nHHDPCs). Cell viability and cell cycle analyses demonstrated that PPD exhibited a significant cytotoxic effect on nHHDPCs through inducing cell death and G_2_ phase cell cycle arrest in a dose-dependent manner. It was additionally observed that treatment of nHHDPCs with PPD induced cellular senescence by promoting cellular oxidative stress. In addition, the results of the current study indicated that these PPD-mediated effects were involved in the alteration of miRNA expression profiles. Treatment of nHHDPCs with PPD altered the expression levels of 74 miRNAs by ≥2-fold (16 upregulated and 58 downregulated miRNAs). Further bioinformatics analysis determined that these identified miRNA target genes were likely to be involved in cell growth, cell cycle arrest, cell death, senescence and the induction of oxidative stress. In conclusion, the observations of the current study suggested that PPD was able to induce several cytotoxic effects through alteration of miRNA expression levels in nHHDPCs.

## Introduction

*Para*-phenylenediamine (PPD), also known as 1,4-diaminobenzene, is a key primary precursor of the oxidative dyes used in hair coloring and tattoos (1,2). However, accumulating evidence has suggested that this compound sensitizes skin to allergic reactions (3,4). Further studies have identified that these allergic reactions predominantly occur due to PPD-mediated activation of dendritic cells (5). Additionally, PPD is a potential carcinogen, which was reported to increase the risk of tumorigenesis in the kidney, liver, thyroid gland and urinary bladder in mice and rats (6,7). In addition, *in vitro* studies have demonstrated that PPD induced reactive oxygen species (ROS)-mediated DNA damage in uroepithelial cells and activated p38 mitogen-activated protein kinase (MAPK) and c-Jun N-terminal kinase in Chang liver cells (8,9). A case report demonstrated that PPD induced severe acute hair loss in females within six days of application (10). However, it remains to be elucidated whether PPD contributes to hair loss by inducing damage to normal human hair dermal papilla cells (nHHDPCs).

MicroRNAs (miRNAs) are small (18–24 nucleotides) noncoding RNAs that repress the translation of target genes through imperfect base pairing to the 3′-untranslated region of their target mRNAs (11,12). miRNAs have been reported to be key regulators of apoptosis, proliferation and differentiation (13). Regarding the role of miRNA in hair, it has been reported that the expression levels of miRNA-31 (miR-31) were upregulated in the anagen phase of the hair growth cycle and controlled the expression levels of Krt16, Krt17, Dlx3 and Fgf10 (14). In addition, miR-24 was reported to regulate the development of hair follicles by targeting the hair keratinocyte stemness regulator Tcf-3 (15). Furthermore, a previous study demonstrated that Dicer, an miRNA-processing enzyme, was essential for the morphogenesis of hair follicles (16).

The aim of the current study was to investigate the effects of PPD on cell growth, death and senescence in nHHDPCs. In addition, the role of PPD in the regulation of the expression profile and the mechanisms of specific miRNAs was evaluated using bioinformatics analysis.

## Materials and methods

### Cells and culture conditions

nHHDPCs (Innoprot, Biscay, Spain) were cultured in Dulbecco’s modified Eagle’s medium (Gibco Life Technologies, Grand Island, NY, USA) supplemented with 10% fetal bovine serum (Gibco Life Technologies) and 1% penicillin-streptomycin (Gibco Life Technologies) at 37°C in an atmosphere of 5% CO_2_. PPD was purchased from Sigma-Aldrich (St. Louis, MO, USA).

### Cell viability assay

Cell viability was monitored using the water-soluble tetrazolium salt (WST-1) assay (EZ-Cytox Cell Viability Assay kit; ITSbio, Seoul, Korea). A total of 5×10^3^ nHHDPCs were seeded into 96-well plates and treated with various concentrations of PPD (0, 100, 200, 300, 400, 500 and 600 *μ*M) for 24 h. Following treatment, nHHDPCs were mixed with 10 *μ*l WST-1 solution and incubated at 37°C for 0.5 h. Cell viability was then determined by measuring absorbance at 450 nm using an iMark plate reader (Bio-Rad Laboratories, Inc., Hercules, CA, USA).

### Propidium iodide (PI)-based cell cycle analysis

The cell cycle was analyzed using PI (Sigma-Aldrich) staining of DNA. nHHDPCs were plated and treated with various concentrations of PPD (0, 200, 400 and 600 *μ*M) for 24 h. Cells were then trypsinized (using 0.25% trypsin-EDTA; Gibco Life Technologies), centrifuged (3,500 × g, 2 min), washed with phosphate-buffered saline (PBS; Gibco Life Technologies) and fixed in 70% ethanol (Merck Millipore, Darmstadt, Germany) at 4°C for 3 h. The fixed cells were incubated with staining solution [50 *μ*g/ml PI, 0.1 *μ*g/ml RNase (Life Technologies, Grand Island, NY, USA) and 0.05% Triton X-100 (Sigma-Aldrich) in PBS] at 37°C for 1 h and then analyzed using a FACSCalibur flow cytometer (BD Biosciences, San Jose, CA, USA). The mean PI fluorescence intensity was determined based on analysis of 10,000 cells using the FLH-2 detection channel (585±42 nm).

### Detection of cellular senescence

For the assessment of cellular senescence, nHHDPCs (2×10^6^) were seeded into 60-mm cell culture dishes and treated with 0 or 400 *μ*M PPD. Following 48 h of treatment, cells were fixed using Fixative solution (included in Senescence Detection kit; BioVision, Inc., Milpitas, CA, USA) and senescence-associated-β-galactosidase (SA-β-gal) activity was measured using the Staining Solution Mix, including Staining Solution, Staining Supplements and X-gal substrate for (SA-β-gal) within the Senescence Detection kit, according to the manufacturer’s instructions. Cells stained for SA-β-gal were counted under a light microscope (CKX41; Olympus Corporation, Tokyo, USA) (magnification, ×200) and the percentage of SA-β-gal positive cells were calculated.

### Detection of intracellular ROS

Staining for ROS in cultured cells was conducted using a 2′,7′-dichlorodihydrofluorescein diacetate (DCF-DA; Sigma-Aldrich) assay. Briefly, 2×10^6^ nHHDPCs were plated in 60-mm culture dishes and treated with PPD. Following treatment for 24 h, the cells were stained by adding DCF-DA to the culture medium to a final concentration of 20 *μ*M and then incubating for 1 h. Distribution of the stained cell population was determined using a FACSCalibur flow cytometer.

### miRNA expression profiling

In order to analyze the miRNA expression profile, nHHDPCs (2×10^6^) were seeded into 60-mm culture dishes and treated with 400 *μ*M PPD. Following 24 h of treatment, total RNA was purified using TRIzol reagent (Life Technologies) according to the manufacturer’s instructions. Total RNA was dephosphorylated and labeled with pCp-Cy3 using an Agilent miRNA Labeling kit (Agilent Technologies, Inc., Santa Clara, CA, USA). Labeled RNAs were hybridized using a SurePrint G3 Human v16 miRNA 8×60K microarray (Agilent Technologies, Inc.) at 65°C for 20 h. The miRNA expression profile was digitized using Feature Extraction version 10.7 software (Agilent Technologies). Fold changes in miRNA expression levels were determined using GeneSpring GX software, version 11.5 (Agilent Technologies).

### Prediction of potential target genes of PPD-regulated miRNAs and gene ontology (GO) analysis

Potential target genes of PPD-regulated miRNAs were predicted using the DNA Intelligent Analysis (DIANA) microT-CDS version 5.0 bioinformatics tool (http://diana.cslab.ece.ntua.gr/). GO of each putative target gene was identified using the Database for Annotation, Visualization and Integrated Discovery (DAVID) bioinformatics resource, version 6.7 (http://david.abcc.ncifcrf.gov). Target genes were categorized into four GO terms: Aging, skin development, apoptosis and cell proliferation. Furthermore, target gene-associated signaling pathways were determined using the Kyoto Encyclopedia of Genes and Genomes (KEGG) pathway algorithm (http://david.abcc.ncifcrf.gov/summary.jsp) within the DAVID resource.

### Statistical analyses

Values are expressed as the mean ± standard error of the mean of three independent experiments. Statistical significance was determined by Student’s t-test and P<0.05 was considered to indicate a statistically significant difference between values.

## Results

### PPD treatment reduces the proliferation rate of nHHDPCs

**Figure 1:**
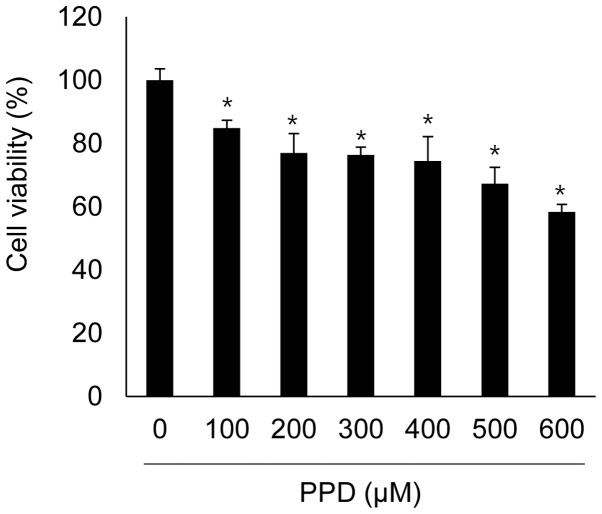
Effect of PPD on viability of nHHDPCs. nHHDPCs (5×10^3^) were seeded into 96-well plates and treated with various concentrations of PPD (0, 100, 200, 300, 400, 500 and 600 *μ*M) for 24 h. Cell viability was measured using the water-soluble tetrazolium salt assay. Values are presented as the mean ± standard error of the mean of the percentage of control optical density of experiments performed in triplicate. ^*^P<0.05 vs. 0 *μ*M PPD. PPD, para-phenylenediamine; nHHDPCs, normal human hair dermal papilla cells.

Previous studies reported that exposure to >250 *μ*M and >60 *μ*M PPD resulted in considerable cytotoxicity in dendritic cells (17) and keratinocytes (18), respectively. Therefore, the current study aimed to determine the cytotoxic effects of PPD on nHHDPCs at concentrations of 0, 100, 200, 300, 400, 500 and 600 *μ*M using a WST-1-based cell viability assay. As shown in Fig. 1, the viability of nHHDPCs was significantly reduced following exposure to PPD for 24 h. Maximal toxicity was observed at 600 *μ*M, at which concentration cell viability was reduced to 58.33±2.39% of the control value (*n*=3; P<0.05). The IC_25_ (a 25% reduction in viability) of PPD was 400 *μ*M, at which concentration cell viability was reduced to 74.42±6.08% of control value (P<0.05) (Fig. 1).

### PPD treatment increases cell death and cell cycle arrest in nHHDPCs

**Figure 2:**
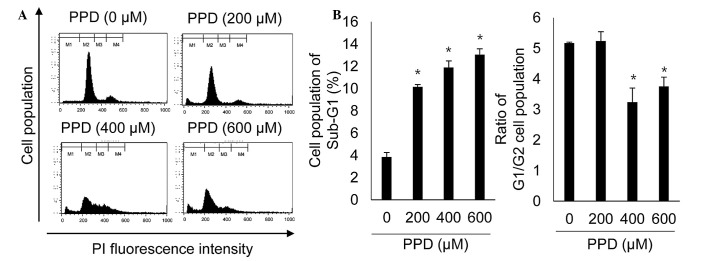
PPD induced cell death and G_2_ arrest in nHHDPCs. nHHDPCs (2×10^6^ cells) were seeded into 60-mm culture dishes, treated with PPD (0, 200, 400 and 600 *μ*M) for 24 h, collected and stained with PI. (A) The fluorescence-intensity distribution of the stained cells was analyzed by flow cytometry. (B) The percentage of sub-G_1_ cells and the ratio of G_1_/G_2_ cells were then quantified. Sub-G_1_, G_1_, S and G_2_/M phases were separated using gates M1, M2, M3 and M4, respectively. ^*^P<0.05 vs. 0 *μ*M PPD. PPD, para-phenylenediamine; nHHDPCs, normal human hair dermal papilla cells; PI, propidium iodide.

The present study investigated whether PPD-induced loss of cell viability occurred due to cell cycle arrest and cell death. Cells were treated with PPD (0, 200, 400 and 600 *μ*M) for 24 h and the distribution of cells in the different cell cycle phases was analyzed using flow cytometry. As shown in Fig. 2, treatment with 200, 400 and 600 *μ*M PPD led to significant accumulation in the sub-G_1_ phase, compared with that of control DMSO-treated cells (P<0.05). In addition, the proportion of cells in G_1_/G_2_ was significantly reduced by PPD (P<0.05), indicating that PPD increased the G_2_ population. These data therefore demonstrated that PPD induced cell death and G_2_ arrest in nHHDPCs.

### PPD treatment leads to the accumulation of intracellular ROS and senescence-like growth

**Figure 3:**
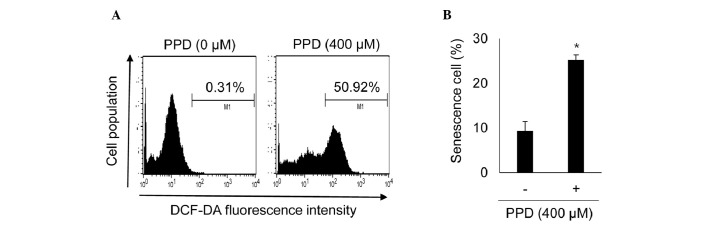
PPD increases intracellular ROS production and senescence in nHHDPCs. (A) nHHDPCs (2×10^6^ cells) were seeded into 60-mm culture dishes and treated with PPD (0 and 400 *μ*M) for 24 h. Cells were then collected and stained with DCF-DA solution. The fluorescence-intensity distribution of DCF-DA-stained cells was analyzed using flow cytometry. Alterations in the intracellular levels of ROS were determined using the M1 gate. (B) Senescence was measured using the senescence associated-β-galactosidase assay. nHHDPCs (2×10^6^) were seeded in 60-mm culture dishes and treated with PPD (0 and 400 *μ*M) for 48 h, fixed and then reacted with X-gal. ^*^P<0.05 vs. 0 *μ*M PPD. PPD, para-phenylenediamine; ROS, reactive oxygen species; nHHDPCs, normal human hair dermal papilla cells; DCF-DA, 2′,7′-dichlorodihydrofluorescein diacetate.

Chye et al (8) observed that PPD increased intracellular ROS and induced apoptosis in Chang normal human liver cells. Therefore, the current study investigated the effects of PPD on the regulation of intracellular ROS production in nHHDPCs. Intracellular ROS levels were determined using an DCF-DA probe, which is oxidized to fluorescent DCF in the presence of ROS. As presented in Fig. 3A, exposure to PPD resulted in a marked increase in fluorescent DCF-positive cells compared with that of the control cells, indicating that PPD increased intracellular ROS production in nHHDPCs. As ROS have been implicated in cellular senescence (19), the present study investigated whether the PPD-mediated increase in ROS was associated with increased senescence by analyzing the activity of SA-β-gal, a marker of cellular senescence. Consistent with the observed ROS increase, PPD was identified to promote an increase in SA-β-gal activity (Fig. 3B), indicating that PPD induces cellular senescence in nHHDPCs.

### Identification of differentially expressed miRNAs in PPD-treated nHHDPCs

Fig. 2 and 3 demonstrated that cell cycle arrest in G_2_ phase and PPD-mediated cell death were characterized by increased ROS production. Therefore, in order to determine whether ROS-mediated cell cycle arrest and cell death are associated with miRNA expression, miRNA microarray analysis was conducted using the SurePrint G3 Human v16 miRNA 8×60K microarray, which contained 2,006 human miRNA probes. Significant miRNAs exhibiting a ≥2.0-fold increase or reduction in expression were selected using GeneSpring GX software. As presented in Table I, PPD differentially regulated the expression levels of 74 miRNAs. Notably, 16 of 74 miRNAs were significantly upregulated and 58 miRNAs were significantly downregulated. In particular, miR-425-3p exhibited the greatest increase in expression (230.60-fold) and miR-3656 the greatest reduction (112.15-fold), compared with the corresponding miRNAs in control cells. These results suggested that the PPD-mediated cellular effects were associated with alterations in expression of specific miRNAs.

### Bioinformatic analysis of PPD-modulated miRNAs

As miRNAs perform their biological functions through regulation of target mRNA translation (11), the present study aimed to predict the target genes of the miRNAs deregulated in response to PPD. The biological functions of the upregulated and downregulated genes were then determined following categorization into the four groups: Aging, apoptosis, cell proliferation and skin development, using DAVID (Tables II and III, respectively). In addition, in order to identify the specific signaling pathways of the deregulated miRNAs, the correlation between KEGG pathway-associated genes and the target genes of each miRNA were analyzed. The meaningful KEGG pathways with a value >1% (percentage of target genes/total genes involved in each pathway) were selected. The analysis identified a wide distribution of cellular functions, which are presented in Tables IV and V. The results indicated that the upregulated miRNAs were implicated in signaling pathways in cancer, ubiquitin-mediated proteolysis, melanogenesis, cell cycle, Wnt signaling, MAPK signaling, neurotrophin signaling, cell adhesion molecules (CAMs), long-term potentiation, natural killer cell-mediated cytotoxicity, calcium signaling, neuroactive ligand-receptor interactions, glycosphingolipid biosynthesis, arrhythmogenic right ventricular cardiomyopathy, axon guidance, ErbB signaling, gonadotropin-releasing hormone signaling, tight junctions and viral myocarditis (Table IV). In addition, PPD-induced downregulated miRNAs were implicated in signaling pathways in cancer, regulation of actin cytoskeleton, Wnt signaling, oocyte meiosis, glycerolipid metabolism, MAPK signaling, insulin signaling, chemokine signaling, cytokine-cytokine receptor interaction, Janus kinase-signal transducer and activator of transcription signaling, calcium signaling, mammalian target of rapamycin signaling, axon guidance, cell cycle, ubiquitin mediated proteolysis, regulation of actin cytoskeleton, ErbB signaling, melanogenesis, TGF-β signaling, vascular smooth muscle contraction, tight junction, neuroactive ligand-receptor interactions, CAMs, glycerophospholipid metabolism, adipocytokine signaling and neurotrophin signaling (Table V).

## Discussion

Although PPD has been widely used in hair dyes and tattoos, previous studies have demonstrated that PPD may be an important etiological factor for allergic contact dermatitis (20,21). However, the side effects of PPD in hair follicle cells remain to be fully elucidated. The results of the current study provided evidence for senescence and cell death as key responses of nHHDPCs to PPD. The senescence-associated alterations observed included G_2_ phase arrest and increases in ROS production as well as SA-β-gal activity. To the best of our knowledge, the present study was to first to report these responses in dermal papilla cells, although PPD-mediated hair loss has been described in a clinical report (10). PPD was reported to promote apoptosis through oxidative stress-induced DNA damage in kidney and liver cells (22,23). The data of the current study confirmed the apoptotic effect of PPD in dermal papilla cells; however, maximal toxicity was obtained at 600 *μ*M, which only increased the proportion of apoptotic cells 9.21%. The most notable observation in the present study was the implication of PPD in the induction of G_2_ phase arrest, cellular ROS production and senescence in dermal papilla cells. The proportion of cells in G_1_/G_2_ phase was significantly reduced at 400 *μ*M, at which concentration ROS production was increased by 50.61% compared with the control group. Consistent with the data described, 400 *μ*M PPD increased the number of senescent cells by 15.90%. It has been previously demonstrated that PPD increased intracellular ROS levels and induced apoptosis in Chang normal human liver cells (8). In the present study, marked alterations in the levels of cell death and senescence were observed in dermal papilla cells following PPD treatment. The data collected indicated that PPD induced G_2_ arrest and ROS production, which in turn triggered cellular senescence leading to cell death in HHDPCs.

Under identical experimental conditions, the present study identified 74 miRNAs that were differentially expressed by ≥2-fold following PPD treatment in nHHDPCs. Among these, 16 miRNAs were significantly upregulated and 58 miRNAs were significantly downregulated in PPD-treated nHHDPCs. Of note, the expression levels of miR-146b-5p were significantly downregulated by 30.17-fold following PPD treatment of the cells. miR-146b-5p has been previously reported to negatively regulate cellular senescence via targeting inter-leukin-1 receptor-associated kinase 1 in fibroblasts (24). In addition, miR-378, which was downregulated by 36.78-fold in the present study, has been previously reported to promote cell survival, tumor growth and angiogenesis through targeting suppressor of fused and fused in sarcoma-1 (25). Together, miR-146b-5p and miR-378 are known to be critical miRNAs involved in cell survival and anti-senescence; thus, regulation of their expression is a promising strategy for the treatment of PPD-mediated cellular senescence in dermal papilla cells. The biological functions of potential target genes of the altered miRNAs were further demonstrated using GO analysis and the web-based program DAVID. The target genes were categorized into four GO terms: Aging, skin development, apoptosis and cell proliferation. Additionally, KEGG pathway analysis identified that the target genes of the miRNAs upregulated by PPD treatment were predominantly implicated in the Wnt and MAPK signaling pathways. The Wnt signaling pathway has been demonstrated to maintain the balance between cell proliferation and differentiation (26). Notably, the Wnt signaling pathway has an important involvement in hair follicle morphogenesis via activation of β-catenin (27). In addition, in bald patients, activation of Wnt signaling was reported to induce reactivation of hair growth (28). Therefore, the results of the KEGG pathway analysis in the present study indicated that PPD regulated hair growth, morphogenesis and proliferation of dermal papilla cells via miRNA-mediated regulation of the Wnt signaling pathway. MAPKs are important intracellular signaling molecules which have pivotal roles in proliferation, differentiation, development, transformation and apoptosis (29). Therefore, the results of the present study suggested that PPD regulated MAPK signaling pathways through altering the expression of specific miRNAs, which in turn altered cell proliferation in dermal papilla cells.

In conclusion, to the best of our knowledge the present study was the first to use cell-based assays and miRNA microarray analysis to demonstrate that PPD significantly induced dermal papilla cell death and senescence through alteration of the expression levels of specific miRNAs. The results of the current study also suggested that the identified miRNAs may be potential candidates for the development of novel treatment strategies for PPD-induced cell dysfunction.

## Figures and Tables

**Table I:** MicroRNAs exhibiting a ≥2-fold alteration in expression following treatment of normal human hair dermal papilla cells with *para*-phenylenediamine.

MicroRNA	Fold change	Direction of regulation
miR-100-3p	57.90	Up
miR-1225-3p	3.43	Up
miR-1228-3p	2.14	Up
miR-1238	2.34	Up
miR-1825	2.08	Up
miR-18b-3p	106.08	Up
miR-191-3p	2.06	Up
miR-3180-5p	100.21	Up
miR-33b-3p	50.55	Up
miR-425-3p	230.60	Up
miR-4286	2.03	Up
miR-4313	2.17	Up
miR-4323	2.02	Up
miR-634	2.26	Up
miR-766-3p	3.78	Up
miR-933	51.08	Up
miR-410	2.48	Down
miR-513a-5p	2.54	Down
miR-500a	2.76	Down
miR-3651	3.07	Down
miR-1207-5p	3.14	Down
miR-762	4.29	Down
miR-150-3p	15.14	Down
let-7a-3p	23.13	Down
miR-1181	110.44	Down
miR-1226-5p	32.72	Down
miR-125a-3p	81.56	Down
miR-128	45.79	Down
miR-134	38.97	Down
miR-138-2-3p	45.91	Down
miR-146b-5p	30.17	Down
miR-148b-3p	83.28	Down
miR-17-3p	58.57	Down
miR-181d	34.46	Down
miR-185-5p	106.57	Down
miR-195-5p	43.11	Down
miR-197-3p	73.04	Down
miR-202-3p	64.16	Down
miR-214-5p	30.65	Down
miR-23a-5p	17.14	Down
miR-28-5p	50.28	Down
miR-301a-3p	45.29	Down
miR-30a-3p	64.02	Down
miR-30e-3p	37.05	Down
miR-324-5p	93.16	Down
miR-342-3p	38.57	Down
miR-3653	31.43	Down
miR-3656	112.15	Down
miR-3663-3p	89.50	Down
miR-369-3p	45.40	Down
miR-369-5p	42.07	Down
miR-370	43.81	Down
miR-371a-5p	15.21	Down
miR-378	36.78	Down
miR-3926	30.72	Down
miR-409-5p	76.96	Down
miR-423-5p	58.63	Down
miR-4271	84.01	Down
miR-4291	96.44	Down
miR-431-5p	50.91	Down
miR-431-3p	93.55	Down
miR-4317	53.97	Down
miR-487a	35.23	Down
miR-501-5p	38.33	Down
miR-505-3p	30.72	Down
miR-513b	46.33	Down
miR-539-5p	42.35	Down
miR-548c-3p	19.22	Down
miR-572	29.95	Down
miR-642b-3p	68.28	Down
miR-650	22.21	Down
miR-660-5p	38.17	Down
miR-770-5p	55.39	Down
miR-874	53.92	Down

miR, microRNA.

**Table II:** Predicted targets of microRNAs upregulated in response to *para*-phenylenediamine treatment in normal human hair dermal papilla cells.

MicroRNA	Biological processes and target genes			
	Aging	Skin development	Apoptosis	Cell proliferation
has-miR-100-3p	RPS6KB1, SLC1A2, RTN4	LEF1, GNAS	NEUROD1, HIPK1, MDM2, RTN4, LEF1, SIX1, RILPL1, SKP2, RPS6KB1, GSDMA, PHF17, RTN3, MAP3K7, SON, MAPK31, ESPL1	LEF1, SIX1, MDM2, SAV1, BHLH41, AKIRN2, PDSSB, ODC1, SKP2, HIPK1, E2F3, RPOX1, NFIB, IRF2, TOB1
has-miR-1225-3p	HMGA1, SREBF2, P2RY2	–	EGLN3, LRP5, ESR1, BIRC5, CASP5, MEF2D, MAX, BMF, IL2, CLN3, AR, MAP3K1, IL2RB	KRT4, TGFBI, CD274, HDGF, EGLN3, LRP5, ESR1, BIRC5, IL2, IGFBP6, AR, RAPGEF3, CD34, GDF2, GFER
hsa-miR-1228-3p	SLC32A1, AMFR	–	EYA1, UBE2B, TJP1, MOAP1, AGAP2, PRDX2, WT1, BMP7, RNF41, CEBPG, CADM1, USP7	AGAP2, PRDX2, WT1, BMP7, THAP1, TSC1, PDCD1LG2, EYA1, WNT9A, MAPRE2, TNS3, LHX9, STAT6
hsa-miR-1238	MSH6	APC	SIX1, THOC1, ING4, CCAR1, MSH6, APC, CTSH, BRCA1, SLIT3, ERCC3, API5	APC, ING4, CD160, SIX1, SP6, BLM, EPS8, BRCA1, TACC3, NASP, DPP4, CTSH, BCAT1, CNBP, PBX1, PTN, IGFBP5
hsa-miR-1825	SERPINE1, RELA, ILK, LMNA, TERF2	DDR1, COL5A3	TM2D1, ROCK1, FXR1, MAX, SOS2, SERPINE1, RELA, ILK, HELLS, ITCH, SULF1, PKN2, LMNA, TRIM2, PDCD5, CECR2	SERPINE1, RELA, ILK, OSR1, DDR1, JAG1, KIT, SMARCA2, NRP1, PDPN, FANCA, PMP22, ITCH, PPARG, OSR1, TGFB2, ETS1, MAP3K11, PPARG, TIAM1, SULF1, ETS1, TGFB2, HELLS, MAP3K11, ACHE, FZD6, STAT6
hsa-miR-18b-3p	HMGA2, SHC1,	–	HMGA2, ITGB1, VHL, IP6K2, RAD21, DUSP6, RRP8, BCL2L2	HMGA2, SHC1, ITGB1, VHL, ACVRL1, CIAO1, RPRD1B, EMP2 NAMPT
hsa-miR-191-3p	–	–	HOXA5, GLI2	HOXA5, GLI2
hsa-miR-3180-5p	CTNNA1, CTSC, CASP7, PTGS1, DCN, MGEA5	APC	CTNNA1, CTSC, CASP7, APC, GPAM, ERBB4, DLC1, NAE1, HOXA5, NAIP, DDX5, GAS2, BRCA1, HIPK2, SRPK2, NF1, ERBB2, MED1, CD38, STK4, MAP3K9, RBM25, TP53INP1, ROCK1, USP47, WNK3, BAX	HOXA5, NDRG2, KIF14, IRS1, ESRRB, BRCA1, NF1, SRPK2, FER, CTBP1, ST8SIA1, FKTN, PDPN, CD274, NFIB, ERBB2, STK4, GPAM, ERBB4, DLC1, MED1, PTGS1, HIPK2, APC, SESN1, CNBP, DOCK2, CDKN2B, FZD9, CDK13
hsa-miR-33b-3p	RTN4	BCL11B	RTN4, DUSP22, DCUN1D3, BCL11B, PDE5A, FGFR1, UBE2Z, RPS3, URI1	BCL11B, PDE5A, DUSP22, FGFR1. NAMPT, FOXF1, CD274, FEZF1, RPS15A, CHRM3
hsa-miR-425-3p	–	–	–	–
hsa-miR-4286	RELA	COL5A2	RELA, MED1, PTK2, PIM1, RRN3, MAP2K7, AGTR2, PSMD11, DYNLL2	RELA, MED1, PTK2, PIM1, RRN3, DCNT2, FOXO4, ACVR2A
hsa-miR-4313	–	–	TNFSF9, FZD5, JAK3	TNFSF9, FZD5, JAK3, WNT3
hsa-miR-4323	VDR, CTSC, MAP2K1	–	VDR, CTSC, JAK3, PIM1, ID1, TAOK2, GSK3B	VDR, MAP2K1, JAK3, PIM1, TGFB1, GABBR1, MMP14
has-miR-634	EDNRA, TGFBR1, RXRA, FURIN	–	EDNRA, RXRA, TRAF5, HRK, HIPK1, TGFBR1, NF1, RNF41, CXCR4, PDCD6IP, MAP2K4	FURIN, TRAF5, HIPK1, PIM1, EDNRA, CASK, ROGDI, NF1, TGFBR1, RXRA, ABI1
hsa-miR-766-3p	CHEK2, TH, SCAP	–	BDNF, NKX2 5, CUL5, SKP2, ESPL1, SLIT3, CARD8, DPF1, CHEK2, ESR2, MKL1, YARS, XAF1, KDM2B, PLEC	ESR2, KDM2B, BDNF, NKX2 5, CUL5, SKP2, CAS8, STAT6, DOCK2, ABCB1, ERF
hsa-miR-933	–	–	ING4, BDNF, MEF2A	ING4, BDNF

miR, microRNA; hsa, homo sapiens.

**Table III:** Predicted targets of microRNAs downregulated in response to *para*-phenylenediamine treatment in normal human hair dermal papilla cells.

MicroRNA	Biological processes and target genes			
	Aging	Skin development	Apoptosis	Cell proliferation
has-let-7a-3p	CNR1, TFRC, TGFBR1, F3, LRP2, ID2, VCAM1, SLC1A2	TCF7L2, JUP, ITGA2	FOXO1, ITCH, TLR4, PDE5A, TGFBR1, F3, CNR1, TCF7L2, MALT1, SGK3, SOX2, CUL1, RHOA, HIPK2, CUL5, JAK2, IGF1R, MEF2C, LRP6, SMO, SKIL, ECT2, ROCK1, OPA1, ID1, PKN2, RNF34, CREB1, APP, HIP1, PAK2, RAD21, UBE2B, DSG2, SOS2	TLR4, PDE5A, MALT1, SGK3, MEF2C, SOX2, CUL1, IGF1R, CDC42, PLAG1, HHIP, NRP3, ID4, CDC73, NR5A2, PROX1, TOB1, RAX6, SNAI2, NR2F2, SALL1, NRP2, UTP20, EMP2, LRP6, SMO, PDSSB, MTSS1, TGFBR1, F3, VCAM1, LRP2, ID2, TCF7L2, RHOA, HIPK2, CUL5, JAK2, FOXO1, ITCH, TCF3, JAG1, FZD3
hsa-miR-150-3p	TFCP2L1	–	INHBA, IL1A, RHOA, ATG7, BCL3, MECOM, CRH, MPO, MAP3K5, NF1, ARHGEF7	RHOA, NF1, PHOX2B, CD164, INHBA, IL1A, MECOM, EVI5, TIMELESS, FER, NDN, FYN, NRP1, BTRC
hsa-miR-1181	–	–	–	–
hsa-miR-1207-5p	RXRA, TERF1, WNT16, TMEM115, GNAO1	DHCR24, TFAP2A, ATP7A	EDAR, ACAA2, GDNF, ALX4 DHCR24, TFAP2A, MAP2K5, PDPK1, ATG7, PACS2, TRIO, RXRA, PIM1, MSX1, IGF1R, ERBB2, PDE1B, BIRC6, CDH1, TERF1, APBB1	MAFG, FOXO4, PBX1, RARA, ERBB2, RAC2, CSF1, MXD4, RXRA, DHCR24, TMEM115, IGF1R, PIM1, MSX1, BIRC6, TFAP2A, WNT16, MAP2K5, TSC2, TENC1, STAT6, MGFEB, SIX5
hsa-miR-1226-5p	TBX3, EDNRA	DDR1	GLI2, DICER1, NF1, MLLT11, TBX3, EDNRA, PTK2, ING4, SKP2, RTN3, SORT1, PEG10, BCL2L2, EIF2AK3	GLI2, DICER1, NF1, NACC2, IL9R, EMX2, HOXC10, SIX2, TBX3, EDNRA, PTK2, ING4, SKP2, SIX1, DDR1, TENC1, MLL2, FKTN
hsa-miR-125a-3p	ULK3	–	RHOA, TAOK1	RHOA, TAOK1, TIRAP
hsa-miR-128	EDNRA, MET, CNR1, SIRT1, F3, MAPK14, MNT, MME, TGFBR1, GRB2	APC, NGFR, COL5A1	NGFR, PIK3R1, HIPK2, BMI1, CITED2, ERCC1, PHB, SIRT1, TGFBR1, CYLD, SLIT1, APC, DAPK1, SOS1, BTG2, CASP8, MAP2K4, MAGI3, PDPK1, MNT, CNR1, MAPK14, FOXO1, NTRK2, TRIM32, MCF2L, WNK3, FOXQ1, PLK2, GSK3B, BCL3	EIF2S2, FOXO4, NRP2, GAB1, SIRT1, TGFBR1, EDNRA, F3, ERCC1, PHB, CITED2, BTG2, RUNX1, SOX11, IRS1, TSC1, NRG1, ASPH, FOXP2, ZEB1, PRKX, JAG1, RAP1B, FZD3, HGF, HIPK2, FOXO1, BMI1, NTRK2, TRIM32, ADAM10, MNT, APC, NGFR, PIK3R1, FBXW7, VEFGC, EBI3
hsa-miR-134	EDN1, CISD2	COL5A2	CXCR2, PAWR, TCHP, BDNF, EDN1, YAP1, STAT5B, PAX8, HIPK2, PDCD7, BARD1, SMAD6, CREB1, ITSN1	EDN1, YAP1, CXCR2, PAWR, CDK13, PKD2, OSMR, KRAS, HIPK2, BDNF, ASCC3, XCL1, STAT5B, MAGI2
has-miR-138-2-3p	RPS6KB1, HMGA2, CHEK2, PNPT1	CTNNB1, APC, SHH	CHEK2, APC, NF1, TP53INP1, FOXO3, MEF2C, SIX1, POLB, HIPK2, SHH, BCL2L1, SLIT3, HMGA2, HMGA2, RPS6KB1, BECN1, PEG3, RNF152, GSK3B	NF1, HIPK2, BCL2L1, BECN1, HMGA2, HMGA2, APC, SHH, FOXO3, MEF2C, SIX1, TOB1, OTP, FER, FRK, PGR, WNT2, LHX9, CD34, E2F3, POU3F2, FGF5, PAX6, KLF5, PLAG1, STAT3, MBD2, CDK13
hsa-miR-146b-5p	NOX4, SMC5, CCL5, LRP2, ATR	APC, ERRFI1, COL4A3	CCL5, APC, ADAM17, CCK, COL4A3, ROBO1, SORT1, SIAH2, TRAF6	CCK, ERBB4, ROBO1, FZD3, CCL5, LRP2, NOX4, APC, ADAM17, CD80, HHIP, NRAS, SMAD4, NUMB
hsa-miR-148b-3p	PTEN, RTN4, SCAP, UCP3, SERPINE1, MNT, IL15, LRP2, CANX	APC, ATP7A, COL2A1	PPARG, MDM4, MITF, USP7, SULF1, IGF1, ROBO1, SOS1, ETS1, ROCK1, HIPK3, MAX, PTEN, SERPINE1, CDKN1B, MNT, RTN4, APC, COL2A1, SIK1	PTEN, SERPINE1, MNT, IL15, MITF, CDKN1B, E2F7, NRAS, APC, PPARG, SULF1, MDM4, LRP2, ETS1, IGF1, ROBO1, FLT1, MXD1, CDK13
hsa-miR-17-3p	PTEN, SERP1, TGFBR1, HMGA2, ICAM1, DLD	BCL11B,	PTEN, TGFBR1, BCL6, BDNF, HMGA2, BCL11B, MAPK1, PPARG, PIK3CA, TRIM2, MEF2C, DIDER, TOPOS, WNK3, TIA1	PTEN, TGFBR1, BCL6, BDNF, HMGA2, BCL11B, MAPK1, MEF2C, DIDER, TOPOS, PPARG, TSC1, NF1B
hsa-miR-181d	PTEN, ATM, RPS6KB1, VCAM, TIMP3, MME	–	RPS6KB1, PTEN, ATM, CUL5, NOTCH2, GATA6, BCL6, BIRC6, CREB1	RPS6KB1, PTEN, ATM, CUL5, NOTCH2, GATA6, BCL6, BIRC6, RUNX1, VIP
hsa-miR-185-5p	RELA, CDK6, MAPK14, ACAN	CDSN, EDA	RELA, MAPK14, BRCA1, AR, MAP2K6, MED1, ERCC3, APP, PAK6, BCL2L2	RELA, CDK6, MED1, BRCA1, AR, CDK2, BAI1, INSIG1
hsa-miR-195-5p	BCL2, PDCD4, MAP2K1, MNT, DLD	WNT7A, ATP7A, EDA	MNT, BCL2, PDCD4, WNT7A, NOTCH2, IGF1R, VEGFA, FOXO1, RAF1, BCL2L2, BFAR, SIAH1	RAF1, CCND1, FBXW7, E2F3, MNT, BCL2, MAP2K1, IGF1R, HMGA1, WNT7A, NOTCH2, VEGFA, FOXO1, ABCB1, FKBP1B
hsa-miR-197-3p	TERT	DDR1	TERT, BRCA1, MAPK7, ING3, PSMD1, MEF2A	BRCA1, DDR1, FTO, LHX9, ASCC3, PDGFRA, INSL4
hsa-miR-202-3p	NOX4	TFAP2B, ERRFI1	TRAF5, YAP1, MEF2C, RAG1, DICER1, DUSP1, TRAF6	NOX4, TFAP2B, DICER1, TRAF5, YAP1, MEF2C, GPC3
hsa-miR-214-5p	CDK6, KL, SLC1A2, TERF2	BCL11B, ERRFI1	BCL11B, COL18A1, OSR1, RERP, E2F2, TP53I3	CDK6, BCL11B, COL18A1, OSR1, DISC1, ABCB1, SMAD4, KLF5
hsa-miR-23a-5p	P2RY2, DCN	TFAP2C, WNT10A	HSPA9, CLIP3, CARD8, NMT1	TLR4, FOXC1, ADAM10, CSF1, MAPRE1, NCK2
hsa-miR-28-5p	–	APC, CDSN	APC, NF1, BAG1, WNK3	APC, NF1, SESN1, TRIM27
hsa-miR-301a-3p	PTEN, EDN1, CDKN1A, LRP2, MET CANX, UCP3	PTGES3, EDA	IRF1, PRNP, RUNX3, ROBO1, SIK1, RAG1, ZMAT3, TRIM2, SULF1, USP28, SOS2, PAK6, EDN1, TGFA, MDM4, E2F2, PTEN, CDK5R1, CDKN1A, USP47, ROCK1, ROBO2	ROBO1, SULF1, USP28, TSC1, MDM4, PRNP, RUNX3, LIPA, MET, IRF1, TGFA, CDK5R1, WNT2B, HOXA3, RBFOX2, CDKN1A, PTEN, EDN1, E2F7
hsa-miR-30a-3p	CACYBP, HMGA2,	–	PTEN, HMGA2, CDKN1B, MEF2C, BIRC6, TRIM2,	CDKN1B, MEF2C, CREBBP, BLM, BIRC6, SUZ12,
hsa-miR-30e-3p	PTEN	–	CREB1, AKAP13, SOS1	PTEN, MXD1, ODZ1, HMGA2
hsa-miR-324-5p	MSH6	–	MSH6, VDAC1, PSME3	CTLA4, FYN, PBX1
hsa-miR-342-3p	ADRB1, EDRNA, BCL2, CASP2	SOX21, EDA, COL5A2, COL1A2, TCF15	BRCA1, BCL6, BCL2L1, E2F1, BCL2, EDNRA, DAPK1, TIA1, NOTCH2, CASP2, ERBB4, PAK2, DUSP6, DRAM1	BCL6, BCL2L1, E2F1, ERBB4, BCL2, NOTCH2, IRAK4, ID4, EDNRA, BRCA1, TACC1, CSF1, PGR, KAT2B
hsa-miR-3651	MSH6	–	MSH6, KLHL20, HIPK1	HIPK1, PRMT5
hsa-miR-3653	HMGA2, PTEN, SOCS2	TFAP2C	PTEN, HMGA2, SOCS2, MEF2C, SORT1, CREB	PTEN, HMGA2, ADAM10, MEF2C, TFAP2C, RUNX1
hsa-miR-3656	–	–	–	–
hsa-miR-3663-3p	FAS, CASP2, CDKN1A, SREBF1		BCL11B, APC, ADAMTS2, COL1A1, COL3A1 CASP2, CDKN1A, DUSP2, COMP	BCL11B, APC, USP28, DSG2, MEF2D, FAS, ARAF, PTH1R, APC, TGFB2, FABP1, CDKN1A, FAS, LIPG, CD80, BCL11B, USP28, DBN1, VSIG4, IL20RB
hsa-miR-369-3p	SIRT1, WRN, MAP2K1, NUAK1, BRCA2, ADH5	BCL11B, ATP7A,	HDAC2, WRN, WNT5A, AHR, CARD11, JAK2, BMP2, XIAP, SATB1, PAWR, SOX2, FGF2, SOS1, OPA1, NDNF, MEF2D, SOX4, HGF, BIRC3, SLIT3, BRCA2, SIRT1, BCL11B, RASSF6, NEUROD1	USP8, CEBPA, ODZ1, PROX1, CARD11, JAK2, BMP2, XIAP, MEGF10, FGF5, ZEB1, PAX6, SATB1, PAWR, SOX2, FGF2, SIRT1, MAP2K1, ADAM10, WNT5A, SOX4, AHR, HGF, NUAK1, BRCA2, HDAC2, CD47, ZEB2, VEGFC, WNT3
hsa-miR-369-5p	–	–	–	–
hsa-miR-370	PNPT1	APC	APC, SMO, RAF1, CCL21, BFAR, PIK3CA, AKAP13	APC, SMO, RAF1, PRTFDC1, FGF7, SMARCD3, CASK, SBDS, RXRB
hsa-miR-371a-5p	–	LEF1, ATP7A	LEF1, STK4, CITED2, BARD1	LEF1, STK4, SOX2, COL8A1
hsa-miR-378a-3p	–	–	MNAT1, IGF1R, SKP2, NAE1	MNAT1, IGF1R, SKP2, TOB2
hsa-miR-3926	INO80C	–	TMX1, BIRC6, SATB1, IGF1R, CKAP2,	TMX1, BIRC6, SATB1, IGF1R, CDK7, WDR6, ABCB1, ARNT
hsa-miR-409-5p	–	–	CREB1, NAIP	UBE2L3, FGFR1OP
hsa-miR-410	HMGA2, DCN, TOP2A, EDN1, SOCS3, CDK1, PTEN, ADM, LRP2, SMC5	–	BAG1, ELMO2, AHI1, MAGI3, HMGA2, EDN1 PTEN, SAV1, CD38, TIAL1, MDM2, BMP2, XIAP1, TBX5, FGF2, ARAF1, TOP2A, ETS1, BDNF, PTK2, PEG3, SNAI1	HMGA2, CDK1, EDN1, PTEN, BDNF, PTK2, TIAL1, MDM2, COL4A3, NBN, XIAP, YAP2, ADM, LRP2, WNT16, EST1, ISL1, NUMB, TOB1, PEX2, E2F7, CBLB, FGF9, KLF5, IRS1, STAT3, LIFR, FST
hsa-miR-423-5p	MSH6, GSN, PML, ILK, CASP2	–	CASP2, PML, ILK, APBB1, MSH6, GSN, LRP5, BAG1	PML, ILK, PTH1R, LRP5, BAP1, KDM4C, ELF4
hsa-miR-4271	HMGA1, SLC6A3, AMFR	–	FOXO3, EI24, MEF2D, MAPT, SPN, CBX4, WNT7B, DAPL1, MAPK1, HMOX1, THRA	WNT7B, FOXO3, SPN, MBD2, HMGA1, MAPK1, HMOX1, MXD11, MLL2, PDGFB, FOXO4, COL4A3BP
hsa-miR-4291	VDR	–	VDR, PIM1, DDX5, CREB1	VDR, PIM1, PGF, IRS1, MCC
hsa-miR-431-5p	CANX	TCF7L1	TNFSF8, SOX9, IRS1, HIPK3	TNFSF8, SOX9, IRS2, NKX6 1
hsa-miR-431-3p	–	–	–	–
hsa-miR-4317	CTNNA1	APC	CTNNA1, APC, IRS2	CTNNA1, APC, IRS2, ESR1
hsa-miR-487a	SIRT1, CNR1, MARCH5	–	SIRT1, CNR1, TMX1, SGK3 BMI1, SGK3	SIRT1, TMX1,
hsa-miR-500a	MORC3	–	AHR, BTG1, SULF1, HDAC2, ERBB3, SRGN, GRID2	MORC3, AHR, BTG1, HDAC1, ERBB3, EMP1, SOX11, PRG4, FGF9, PEX2, AKIRIN2, BLM
hsa-miR-501-5p	NR3C1, MAP2K1, TFRC	–	NR3C1, CUL1, MEF2C, BMI1, ING3, TMBIM4, TRIM39	NR3C1, CUL1, MEF2C, BMI1, MAP2K1, BCAT1, NOX1
hsa-miR-505-3p	CHEK1	–	TBX5, HMGB1	TBX5, ATP8A2, IL11
hsa-miR-513a-5p	PRKCD, NEK6, MORC3, MAP2K1, CDK6, ACAN, SERP1	APC, SFN, WNT7A, TFAP2B	PRKCD, NEK6, APC, WNT7A, GATA3, XIAP, STAT1, MITF, BCL6, PPARG, ISL1, GZMB, MED1, HDAC2, SFN, EYA1, TRIO, SOS1, ECE1, VAV2, USP47, HGF, BCL2L11, WNK3, OCLN, MAL	PRKCD, MORC3, CDK6, APC, SFN, WNT7A, TFAP2B, BCL6, PCM1, RXRB, PDXK, FOXP2, GATA3, STAT1, MITF, BTG1, HDAC2, XIAP, ISL1, MAGI2, MAP2K1, XRCC5, PPARG, E2F7, NFIB, VIP, ATF3, PURA, CSF1, KRAS
has-miR-513b	TGFBR1, ID2, SIRT1, RNT4, ADH5	BCL11B, TFAP2A, ATP7A	BCL2L1, PPARG, BMF, PAK6, TGFBR1, SIRT1, RNT4, PAK1, SNAI2, BCL11B, MOAP1, TCF7L2, ERBB4, XIAP, CREB1, API5, BCL2L11	TGFBR1, SIRT1, MARCKSL1, SNAI2, ERBB4, PAK1, XIAP, LIFR, FER, BCL11B, AGTR1, GATA2, VIP, BCL2L1, IRF2, ID2, FOXP2, WDR6, VTI1B
hsa-miR-539-5p	SMC5, SLC1A2	TCF7L2, TFAP2A	CUL2, ERBB4, ELMO2, SET, TCF7L2, TFAP2A, HDAC2, TRIM2, PPARGC1A, SIX4	ERBB4, FRS2, HDAC2, PBX1, TCF7L2, CUL2, CD47, DAB2, TFAP2A, VAX1, FKTN
hsa-miR-548c-3p	NR3C1, PTEN, MORC3, SMC5	SHH, BCL11B, TCF7L2	MITF, ERBB4, CITED2, XIAP, SGK3, TAOK1, RAG1, HIPK3, NR3C1, PTEN, SHH, TCF7L2, BTG1, LRP6, GATA6, REST, TIA1	GATA6, REST, ERBB4, SGK3, NR3C1, PTEN, MORC3, SHH, ADAM10, FER, HHIP, KRAS, TCF7L2, BTG1, LRP6, MITF, CITED2, XIAP, SSR1, EREG, SOX11, RUNX1, PURA, E2F3
hsa-miR-572	–	–	–	–
hsa-miR-642b-3p	HMGA2, MET, CDKN1A, PTEN	BCL11B	BCL11B, PDCD10, WT1, RB1, HMGA2, PTEN, CDKN1A, EIF2AK2, IFNG, MAPK8	BCL11B, PDCD10, WT1, RB1, CDKN1A, NR2F2, PROX1, PTEN, EIF2AK2, HMGA2, PAX6, AMBN, PHOX2B
hsa-miR-650	MADCAM1, SHC1	–	LRP6, MITF, PIM1, CUL2, JAK3, MUL1, CSTB, AXL	SHC1, LRP6, MITF, PIM1, CUL2, JAK3, ERF, FTO
hsa-miR-660-5p	–	TFAP2B	TFAP2B, BRCA1, HIPK1	TFAP2B, BRCA1, HIPK1, SF1
hsa-miR-762	RELA, PML	–	RELA, PML, MAPK1, MEN1, PAK4, ITCH, BCL6, PPARD, ADD1, PAX7, ITGB2, MYO18A	RELA, PML, MAPK1, MEN1, PAK4, ITCH, BCL6, PPARD, MMP14, BAP1, FTO, WAR5, FSCN1, XIRP1. TENC1
hsa-miR-770-5p	HMGA2, CNR1	RYR1	HMGA2, CNR1, MED1, SGK3, XAF1, HELLS, MAP3K1	HMGA2, BTG1, LRP6, SGK3, HELLS, CCND2, MED1, PBX1, TSG101, NFIB
hsa-miR-874	DDC	–	ESR1, HIPK2, PAK7, THRA, SORT1	RXRB, COMT, BMRP2, NPR1, ESR1, HIPK2, PAK7, CD276, MXI1

miR, microRNA; hsa, homo sapiens.

**Table IV:** Main functions of upregulated microRNAs predicted using bioinformatics analysis.

MicroRNA	Putative target genes	KEGG pathway	Genes involved in the term	% involved genes	P-value
has-miR-100-3p	167	Pathways in cancer	7	4.2	1.10E-02
		Ubiquitin mediated proteolysis	5	3.0	8.00E-03
		Melanogenesis	4	2.4	2.00E-02
		Cell cycle	4	2.4	3.60E-02
		Wnt signaling pathway	4	2.4	5.80E-02
hsa-miR-1225-3p	183	MAPK signaling pathway	7	3.8	6.20E-02
		Neurotrophin signaling pathway	5	2.7	4.20E-02
		Cell adhesion molecules	5	2.7	5.10E-02
hsa-miR-1228-3p	198	Wnt signaling pathway	4	2.0	1.30E-01
		MAPK signaling pathway	4	2.0	3.80E-01
hsa miR-1238	130	–	–	–	–
hsa miR-1825	321	Pathways in cancer	9	2.8	6.90E-02
		MAPK signaling pathway	8	2.5	6.40E-02
hsa-miR-18b-3p	108	Long term potentiation	4	3.7	3.80E-03
		Natural killer cell mediated cytotoxicity	4	3.7	2.40E-02
		Calcium signaling pathway	4	3.7	4.80E-02
hsa-miR-191-3p	11	–	–	–	–
hsa-miR-3180-5p	489	–	–	–	–
hsa-miR-33b-3p	121	Neuroactive ligand receptor interaction	5	4.1	6.10E-02
		Calcium signaling pathway	4	3.3	8.40E-02
hsa-miR-425-3p	8	–	–	–	–
hsa-miR-4286	87	Glycosphingolipid biosynthesis	2	2.3	6.30E-02
hsa-miR-4313	57	Arrhythmogenic right ventricular cardiomyopathy	3	5.3	2.60E-02
		Melanogenesis	3	5.3	4.20E-02
		Wnt signaling pathway	3	5.3	8.90E-02
hsa-miR-4323	153	Axon guidance	5	3.3	2.10E-02
		ErbB signaling pathway	4	2.6	3.40E-02
		GnRH signaling pathway	4	2.6	4.60E-02
hsa-miR-634	207	GnRH signaling pathway	5	2.4	4.10E-02
hsa-miR-766-3p	357	Tight junction	10	2.8	4.60E-04
		Viral myocarditis	8	2.2	2.00E-04
hsa-miR-933	9	–	–	–	–

miR, microRNA; hsa, homo sapiens; KEGG, Kyoto Encyclopedia of Genes and Genomes; MAPK, mitogen-activated protein kinase; GnRH, gonadotropin-releasing hormone.

**Table V:** Main functions of downregulated microRNAs predicted using bioinformatics analysis.

MicroRNA	Putative target genes	KEGG pathway	Genes involved in the term	% involved genes	P-value
has-let-7a-3p	626	Pathways in cancer	24	3.8	4.60E-04
		Regulation of actin cytoskeleton	15	2.4	1.20E-02
		Wnt signaling pathway	13	2.1	4.20E-03
has-miR-150-3p	184	Wnt signaling pathway	5	2.7	6.00E-02
		Oocyte meiosis	4	2.2	9.30E-02
has-miR-1181	2	–	–	–	–
has-miR-1207-5p	503	Regulation of actin cytoskeleton,	11	2.2	2.50E-02
		MAPK signaling pathway	11	2.2	8.60E-02
has-miR-1226-5p	219	–	–	–	–
has-miR-125a-3p	42	Glycerolipid metabolism	2	4.8	7.70E-02
has-miR-128	642	MAPK signaling pathway	22	3.4	1.50E-04
		Insulin signaling pathway	13	2.0	1.50E-03
has-miR-134	245	Chemokine signaling pathway	7	2.9	1.70E-02
		Cytokine-cytokine receptor interaction	7	2.9	7.10E-02
		Jak-STAT signaling pathway	6	2.4	2.90E-02
		Calcium signaling pathway	6	2.4	4.60E-02
has-miR-138-2-3p	345	Pathways in cancer	14	4.1	3.90E-03
		MAPK signaling pathway	11	3.2	1.60E-02
has-miR-146b-5p	314	–	–	–	–
has-miR-148b-3p	454	Pathways in cancer	20	4.4	5.30E-04
has-miR-17-3p	307	MAPK signaling pathway	10	3.3	1.30E-02
		Pathways in cancer	9	2.9	9.60E-02
		Insulin signaling pathway	7	2.3	1.20E-02
		mTOR signaling pathway	6	2.0	8.20E-04
has-miR-181d	286	Insulin signaling pathway	6	2.1	2.10E-02
has-miR-185-5p	423	Axon guidance	10	2.4	5.70E-04
has-miR-195-5p	506	Pathways in cancer	21	4.2	1.30E-03
		MAPK signaling pathway	14	2.8	4.80E-02
		Wnt signaling pathway	13	2.6	1.40E-03
		Insulin signaling pathway	12	2.4	1.90E-03
		Cell cycle	10	2.0	1.10E-02
		Ubiquitin mediated proteolysis	10	2.0	1.90E-02
has-miR-197-3p	216	Ubiquitin mediated proteolysis	6	2.8	1.10E-02
		Calcium signaling pathway	5	2.3	9.90E-02
has-miR-202-3p	223	Axon guidance	5	2.2	7.80E-02
has-miR-214-5p	196	Regulation of actin cytoskeleton	6	3.1	6.70E-02
has-miR-23a-5p	99	–	–	–	–
has-miR-28-5p	157	MAPK signaling pathway	7	4.5	1.20E-02
has-miR-301a-3p	470	Regulation of actin cytoskeleton	13	2.8	1.30E-02
has-miR-30a-3p	221	Pathways in cancer	9	4.1	3.70E-02
		ErbB signaling pathway	5	2.3	1.90E-02
has-miR-30e-3p	185	Pathways in cancer	8	4.3	5.70E-02
		Jak-STAT signaling pathway	5	2.7	8.10E-02
		ErbB signaling pathway	4	2.2	6.40E-02
		Melanogenesis	4	2.2	8.70E-02
has-miR-324-5p	39	–	–	–	–
has-miR-342-3p	386	Calcium signaling pathway	9	2.3	2.60E-02
		TGF-β signaling pathway	7	1.8	8.00E-03
has has-miR-3651	57	–	–	–	–
has-miR-3653	87	–	–	–	–
has-miR-3656	10	–	–	–	–
has-miR-3663-3p	305	MAPK signaling pathway	12	3.9	5.90E-03
has-miR-369-3p	743	MAPK signaling pathway	15	2.0	9.90E-02
has-miR-369-5p	2	–	–	–	–
has-miR-370	260	Pathways in cancer	8	3.1	6.10E-02
		Chemokine signaling pathway	6	2.3	5.00E-02
has-miR-371a-5p	216	Wnt signaling pathway	5	2.3	6.70E-02
has-miR-378a-3p	101	Pathways in cancer	7	6.9	2.00E-02
has-miR-3926	274	–	–	–	–
has-miR-409-5p	16	–	–	–	–
has-miR-410	852	Pathways in cancer	29	3.4	4.80E-04
		MAPK signaling pathway	20	2.3	2.50E-02
		Wnt signaling pathway	16	2.0	2.50E-03
has-miR-423-5p	218	Calcium signaling pathway	6	2.8	2.20E-02
		MAPK signaling pathway	6	2.8	9.80E-02
		Insulin signaling pathway	5	2.3	3.50E-02
has-miR-4271	247	Chemokine signaling pathway	6	2.4	9.40E-02
has-miR-4291	88	–	–	–	–
has-miR-431-5p	172	Wnt signaling pathway	5	2.9	2.70E-02
has-miR-431-3p	2	–	–	–	–
has-miR-4317	67	Vascular smooth muscle contraction	3	4.5	9.00E-02
has-miR-487a	184	MAPK signaling pathway	7	3.8	4.90E-02
		Tight junction	6	3.3	1.00E-02
has-miR-500a	260	Ubiquitin mediated proteolysis	7	2.0	8.50E-02
has-miR-501-5p	179	Ubiquitin mediated proteolysis	6	3.4	8.20E-03
has-miR-505-3p	30	–	–	–	–
has-miR-513a-5p	773	Pathways in cancer	24	3.1	2.00E-02
		MAPK signaling pathway	20	2.6	2.80E-02
		Focal adhension	18	2.3	7.90E-03
		Regulation of actin cytoskeleton	17	2.2	3.00E-02
has-miR-513b	557	Neuroactive ligand-receptor interaction	12	2.2	8.60E-02
has-miR-539-5p	340	–	–	–	–
has-miR-548c-3p	438	Pathways in cancer	17	3.9	2.20E-03
		Wnt signaling pathway	12	2.7	4.70E-04
		Insulin signaling pathway	9	2.1	9.90E-03
has-miR-572	4	–	–	–	–
has-miR-642b-3p	180	Pathways in cancer	7	3.9	4.60E-02
		Cell adhesion molecules	5	2.8	2.10E-02
has-miR-650	151	Glycerophospholipid metabolism	3	2.0	9.90E-02
has-miR-660-5p	104	Adipocytokine signaling pathway	3	2.9	7.70E-02
has-miR-762	342	Axon guidance	10	2.9	3.80E-04
		MAPK signaling pathway	9	2.6	9.40E-02
		Cell adhesion molecules (CAMs)	7	2.0	2.70E-02
		Cell adhesion molecules (CAMs)	7	2.0	2.70E-02
has-miR-770-5p	171	Neurotrophin signaling pathway	6	3.5	4.00E-03
		MAPK signaling pathway	6	3.5	7.90E-02
has-miR-874	99	–	–	–	–

miR, microRNA; hsa, homo sapiens; KEGG, Kyoto Encyclopedia of Genes and Genomes; MAPK, mitogen-activated protein kinase; Jak-STAT, Janus kinase-signal transducer and activator of transcription; mTOR, mammalian target of rapamycin; TGF-β, transforming growth factor-β.
